# Behaviour change for better health: nutrition, hygiene and sustainability

**DOI:** 10.1186/1471-2458-13-S1-S1

**Published:** 2013-03-21

**Authors:** Rachel S Newson, Rene Lion, Robert J Crawford, Valerie Curtis, Ibrahim Elmadfa, Gerda IJ Feunekes, Cheryl Hicks, Marti van Liere, C Fergus Lowe, Gert W Meijer, BV Pradeep, K Srinath Reddy, Myriam Sidibe, Ricardo Uauy

**Affiliations:** 1 Unilever Research and Development, Olivier van Noortlaan 120, 3133 AT Vlaardingen, the Netherlands; 2 Unilever Research and Development, Quarry Road East, Bebington, Wirral CH63 3JW, UK; 3 London School of Hygiene and Tropical Medicine, Keppel Street, London WC1E 7HT, UK; 4 International Union of Nutritional Sciences, Althanstrasse 14, A-1090 Vienna, Austria; 5 Collaborating Centre on Sustainable Consumption & Production, Hagenauer Strasse 30, 42107 Wuppertal, Germany; 6 Global Alliance for Improved Nutrition, Rue de Vermont 37-39, 1202 Geneva, Switzerland; 7 Bangor University, Gwynedd, Bangor LL57 2DG, UK; 8 Unilever Consumer Marketing Insights, Mapletree Business City, International Business Park, Singapore; 9 Public Health Foundation of India, ISID Campus, 4 Institutional Area, Vasant Kunj, New Delhi 110070, India; 10 Unilever Global Social Mission, 100 Victoria Embankment, London EC4Y 0DY, UK; 11 INTA, University of Chile, Av. El Líbano, 5524 Macul, Santiago, Chile

## Abstract

As the global population grows there is a clear challenge to address the needs of consumers, without depleting natural resources and whilst helping to improve nutrition and hygiene to reduce the growth of noncommunicable diseases. For fast-moving consumer goods companies, like Unilever, this challenge provides a clear opportunity to reshape its business to a model that decouples growth from a negative impact on natural resources and health. However, this change in the business model also requires a change in consumer behaviour. In acknowledgement of this challenge Unilever organised a symposium entitled ‘Behaviour Change for Better Health: Nutrition, Hygiene and Sustainability’. The intention was to discuss how consumers can be motivated to live a more healthy and sustainable lifestlye in today’s environment. This article summarises the main conclusions of the presentations given at the symposium. Three main topics were discussed. In the first session, key experts discussed how demographic changes – particularly in developing and emerging countries – imply the need for consumer behaviour change. The second session focused on the use of behaviour change theory to design, implement and evaluate interventions, and the potential role of (new or reformulated) products as agents of change. In the final session, key issues were discussed regarding the use of collaborations to increase the impact and reach, and to decrease the costs, of interventions. The symposium highlighted a number of key scientific challenges for Unilever and other parties that have set nutrition, hygiene and sustainability as key priorities. The key challenges include: adapting behaviour change approaches to cultures in developing and emerging economies; designing evidence-based behaviour change interventions, in which products can play a key role as agents of change; and scaling up behaviour change activities in cost-effective ways, which requires a new mindset involving public–private partnerships.

## Introduction

The current rate of population growth translates directly into increased numbers of consumers and needs for goods and services. This growth provides consumer goods companies, like Unilever, with opportunities to grow. A key challenge in this progression, however, is to address these needs in a manner that promotes good nutrition, promotes proper hygiene and minimises the impact on the environment (sustainability). Growth following traditional business models is not suited to working with these targets, in both the short term and the longer term. In light of this, Unilever has developed the Unilever Sustainable Living Plan [1] – a set of new, global targets in the areas of improving health and well-being, reducing environmental impact and enhancing livelihoods. This plan includes product reformulations (for example, nutrition: lowering sodium in foods), changes in design of products (for example, sustainability: improved packaging to reduce waste) and consumer behaviour change (for example, hygiene: encouraging handwashing with soap). Analyses of this plan, and current progress, indicate that creating behaviour change amongst the general population is one of the greatest challenges Unilever will face in the execution of the Unilever Sustainable Living Plan. Nutrition, hygiene and sustainable behaviours are the biggest areas where change needs to be achieved. For example, an analysis of the footprint of Unilever products across their lifecycle has indicated that consumer use of the product is responsible for almost 70% of the sustainability footprint.

Historically, the success of private industry has been driven by its ability to influence consumer behaviour and develop new markets. Fast-moving consumer goods companies like Unilever therefore have a clear role to play in making sustainable and healthy living possible (for example, regular handwashing, lowering salt intake, making sustainable choices). However, the scale of this challenge requires a bigger, faster, concerted approach between multiple sectors [2]. Combining academic and public health expertise in conducting evidence-based interventions with industry marketing power and consumer and health understanding will enable the delivery of long-term, practical solutions that will help to address the challenges of achieving behaviour for better health. Examples of alliances such as Water and Sanitation for the Urban Poor and Scaling Up Nutrition highlight where this approach has brought success.

In light of this challenge, Unilever organised a symposium entitled Behaviour Change for Better Health: Nutrition, Hygiene and Sustainability. The aims of the symposium were to discuss the science behind this behaviour change challenge, to identify new directions for evidence-based solutions, and to stimulate new collaborations designed to increase the reach and impact of behaviour change approaches.

The symposium consisted of three sessions of internal and external speakers from a variety of fields and backgrounds. Session 1 detailed challenges in behaviour change for better health, Session 2 explored behaviour change theory and applied best-practice examples, and Session 3 examined collaborations as a key to success in behaviour change. The audience consisted of academics, nongovernmental organisation and business participants from the public and private sectors, as well as Unilever senior researchers and representatives from Unilever’s business functions.

This article gathers the main conclusions of the presentations given at the symposium.

## Session 1: key challenges in behaviour change for better health

The first session set the scene for the symposium by summarising key population trends that affect public health in both developed and developing and emerging markets. These trends were translated into key behaviours that people had to change in order to live more sustainably. Professor K Srinath Reddy set the scene by exploring health transition and the role that behaviour change has to play to counteract some of the negative consequences of this transition. Professor Ricardo Uauy discussed the role of prevention in achieving better health. Cheryl Hicks translated future scenarios for sustainable living into individual targets for change.

### Health transition and behaviour change *Professor K Srinath Reddy*

Health transition is a dynamic process that every society experiences as it evolves on the scale of socioeconomic development. The remarkable pace of global health transition, however, has been most marked over the last half-century, resulting in a recast of public health challenges and a reordering of health system priorities across all regions of the world. Over the course of the 20th century, infectious and nutritional deficiency disorders have yielded place to noncommunicable diseases (NCDs) as the foremost cause of death and disability globally. While this was most evident in high-income countries, the low-income and middle-income countries are also presently experiencing escalating epidemics of NCDs such as cardiovascular diseases, cancers, diabetes, obesity, chronic respiratory disorders and mental illness, even as they are still combating the unconquered threats of infectious diseases and endangered maternal and child health [3,4].

NCDs resulted in 36 million deaths in 2008, accounting for 63% of the global death toll of 57 million that year. Low-income and middle-income countries contributed to 80% of the global NCD deaths and to 90% of the 9 million NCD deaths that occurred below 60 years of age [5]. The consequences of such premature mortality can be catastrophic for global economy, national development and the financial stability of affected individuals and families.

Health transition has several stages and many determinants: demographic, nutritional, economic, sociocultural and ecological changes are among the most important [6]. These determinants operate across society and are increasingly becoming global in their impact, even though the effects of transition may vary across and within populations at any given stage of transition. The dynamics of health transition as an evolutionary process is important to understand, so that we can anticipate and attenuate future epidemics.

The world is witnessing shifts in dietary patterns and levels of physical activity, with increasing overweight and obesity and a rising burden of NCDs. Social determinants lead to unhealthy personal behaviours, which in turn cause biologic perturbations that result in disease. Response to health transition must act across this whole cascade of risk to reduce health burdens. Developments in agriculture, trade and environment are as germane to an understanding of health transition as are the biological dynamics of disease causation and transmission. The spectrum of research on disease causation therefore stretches from molecules to markets.

Behaviour change has often been positioned as a required individual adaptation for avoiding or reducing the risk of ill-health. However, the determinants of health operate at multiple levels: individual, family, community, country and the world. At the level of the individual, there is interplay between beliefs, behaviours and biology that affects the balance between health and disease. At the level of families and communities, cultural perceptions, social and economic priorities and pathways of access to health-promoting environments as well as health services are key determinants of health. At the national and global levels, the stage and speed of development, the distribution of developmental benefits across social groups (equity) and the demand–supply issues of trade act as drivers of health transition at the macro level, impacting downstream on the health and well-being of families and individuals.

Since health transition is increasingly being influenced by upstream determinants (for example, features of the social environment), behaviour change is also needed at the societal level. Policy interventions have been shown to have an important impact on health – for example, the North Karelia project used community and policy interventions to reduce cholesterol in the community, and resulted in a large reduction in rates of cardiovascular disease [7]. However, policy interventions must stimulate and support personal choices for good health, even as education enhances knowledge, alters attitudes and helps people to acquire the skills needed for change. Global evidence suggests that behaviour change is best accomplished when education is accompanied by policies that enable individuals to make and maintain healthy choices across their lifespan [7].

The determinants of NCDs, nutritional disorders and even zoonotic diseases are convergent with those that degrade the environment. Industrial-scale livestock production, for example, not only increases meat consumption to unhealthy levels (with NCDs as the principal consequence) but also establishes a conveyor belt for transmission of microbes from wildlife to the veterinary population and then on to the human host. The industrial-scale production is also responsible for grain diversion (accentuating food insecurity), is water intensive (aggravating water insecurity) and is responsible for large-scale production of methane that contributes to global warming. In turn, environmental degradation affects agriculture, biodiversity and availability of water. We are living in an era where food systems threaten the environment and environmental change threatens the food systems. Similarly, tobacco is not only a threat to health but is also a cause of deforestation and environmental pollution.

The response to health transition therefore has to be positioned in the context of sustainable development. Behaviour change has to occur at the level of persons (individuals), of people (communities) and of populations (nations) so that the health of the planet is also protected. The response to health transition needs to extend from an epidemiological model to an ecological model, if global health has to be protected and promoted in the 21st century.

### Prevention for better health *Professor Ricardo Uauy*

First it must be acknowledged that health is a key public good and a prerequisite for human, social and economic development. The duty of governments worldwide is to act in the interests of all people, especially those most vulnerable: children, women, older people, the impoverished, the diseased and the disabled (considering all stages of the lifecourse).

Prevention of malnutrition in all its forms includes addressing the pending agenda of protein energy (stunting and wasting) and micronutrient (vitamin A, iron and zinc) deficiencies, as well as controlling the progression of the diet and physical activity-related NCDs (obesity, diabetes, cardiovascular disease and cancer). This needs to occur in the context of recognition that protection and preservation of public health is a key priority for government action. Prevention should start as early as possible in life – even before conception – and should be continued throughout pregnancy, infancy and childhood to have the greatest impact.

The global population generally aspires to a better life for themselves and for future generations. To achieve optimal nutrition and keep active lives, as well as what needs to be done to avoid disease and disability, everyone needs to learn to communicate what it is that needs to be done. Education is an important first step to raise awareness, but this is insufficient to change behaviour by itself. Motivational messages tailored to the context of the recipient – addressing their beliefs, their situation and their views of the future – are necessary.

Governments, civil society organisations, development agencies and the global public health community at large should reframe NCD prevention within a strategy that places NCDs as a barrier to development; this means explicitly including them as a target for ‘technical assistance, capacity building, program implementation, impact assessment of development projects, funding, and other activities’, as recommended by the World Health Organization diet and physical activity prevention of chronic disease report [8] and the USA Institute of Medicine [9].

Future Millennium Developmental Goals beyond 2015 should set specific targets that address primordial, primary, secondary and tertiary prevention and access to treatment of NCDs. Governments should generate data from periodic surveys across all age groups to better understand and address current factors that determine the burden of NCDs and long-term effects of NCDs; surveillance should also provide more reliable future projections of the NCD burden. Government and relevant private-sector actors should implement programmes that tackle the social determinants of NCDs with particular reference to the following: access to information on food choices, promotion of healthy diets and active lives, and providing access to preventive guidance and treatment when required. All stakeholders including the private sector should join forces in reducing the amount of salt, sugar and saturated fat content in the food supply and should eliminate trans-fat intake, with an emphasis on minimising impact on prices so that all groups of society benefit from the healthier alternatives.

Civil society organisations, development agencies and the global public health community at large should strengthen maternal and child health programmes to reduce maternal and infant mortality rates as a means to assert women and children’s health rights. Governments and private-sector actors should implement recommendations from the World Health Organization policy guidance Recommendations on the Marketing of Foods and Non-Alcoholic Beverages to Children [10]. Governments should include health across the lifespan as a pillar of all policies to enhance the conditions and health system in which people are born, grow, live, work and age. The protection of the rights and freedoms of citizens includes access to healthy diets and active lives; it requires the wise use of rules and regulations that are equitable and recognise the need to protect and preserve all human rights, including the right to healthy, safe and nutritious foods. Policies and programmes to support behaviour change should be based on the best evidence of actual effectiveness, and ideally cost-effectiveness in achieving the desired change. Furthermore, these policies should promote the view that these diseases should not be considered solely as the fault of the individual, but as societal problems that require societal solutions. Starting early in life is indeed necessary to achieve healthy growth, active lives and eventually achieve reductions in preventable death and disability from NCDs.

### Sustainability, the consumer and lifestyles *Cheryl Hicks*

Average lifestyles in most of the western world exceed sustainable levels by a factor of three to five. The biggest impacts amongst Europeans fall into the categories of food, mobility and housing [11]. Our lifestyle choices are also adversely affecting our health and well-being [12]. Consumption patterns of individuals linked to their choice of lifestyles differ around the world but also from household to household. Enabling and encouraging more sustainable lifestyle models requires a deeper understanding of differing lifestyle needs and desires to be met, and the differing motivators, influencers and triggers to behaviour change. Individual lifestyle choices can be shaped by national policies, cultural norms, availability of resources, goods and services, but also by personal desires and societal trends. Behaviour change can be dependent on a person’s sense of agency and in today’s society is increasingly driven by one’s need for instant gratification, or one’s ability to delay it.

Alternative, and less impactful, models of living are also emerging. Examples of promising practice have been scattered, but they do exist and their numbers are growing. Taken together, these examples provide us with a picture of what more sustainable living practices could look like. Forecast into the future, these alternative living models become signposts to possible futures where current lifestyle impacts have been overcome. The SPREAD Sustainable Lifestyles 2050 Social Platform Project, funded by the European Commission’s Seventh Framework Programme: Socio-economic Sciences and Humanities, has been exploring the key challenges to more sustainable living in Europe, and has created scenarios for more sustainable living in 2050. The five challenges for sustainability, lifestyles and consumers are as follows:

♦ *Challenge 1: translating sustainability into meaning for our daily lives.* Our concept of more sustainable ways of living refers to those practices that have made progress towards the minimisation of current harmful consumption and lifestyle impacts while optimising quality of life and personal well-being. To address the current impacts of unsustainable European lifestyles and consumption patterns, we need to understand more specifically where the most significant impacts occur; what is driving those impacts; where the most significant improvements can be made; as well as the evidence of promising practice already underway demonstrating improvements [13].

♦ *Challenge 2: understanding the impacts of our lifestyles.* The most significant individual lifestyle impacts have been identified in the food we eat, the way we move around via transport, and how we live in our homes in terms of energy and material use. These dominant individual lifestyle and private consumption impacts are referred to as impact hotspots [14]. Individual lifestyle impacts in these three areas account for 65 to 75% of Europe’s environmental impacts [11,14]. For Europeans, meat and dairy consumption accounts for almost one-quarter (24%) of all final consumption impacts – by far the largest share in the food and drink sector. Household energy use – including domestic heating, water consumption, appliance and electronics use – accounts for 40% of Europe’s total energy consumption (with space heating alone accounting for 67% of household energy consumption in the European Union’s 27 Member States) [11]. Car ownership, related to dependency on single-car use, in the European Union’s 27 Member States increased by more than one-third (35%) between 1990 and 2007 [11]. Over one-third of the world’s 750 million automobiles are owned by drivers in the European Union.

♦ *Challenge 3: changing individual and corporate behaviours towards sustainability and at scale.* Change, for individuals and groups, often occurs when significant trauma undermines and demands a reformation of the value system. Recent research in neuroplasticity shows that it is possible to change our behaviours, but old behaviours have to be unlearned first [15]. When behaviours are strongly associated with reward and pleasure in the brain – as is the case for most consumption above subsistence – the unlearning is very difficult because strongly reinforced neuronal connections have to be broken. For change to occur without trauma, we have learned that several key things need to happen: the changes proposed have to satisfy the individual’s needs; old behaviours need to be unlearned; unknown changes need space; positive reinforcement or feedback is critical [16]; and neuroplasticity research also suggests that individuals trying to make changes that are not obviously more pleasurable than the previous options should not try to make more than one kind of change at a time.

♦ *Challenge 4: envisioning a future of more sustainable lifestyles.* To create scenarios for more sustainable lifestyles in future societies, we need to define common targets that will allow us to identify when we have reached our goals for essential sustainable living. The SPREAD project has defined the material footprint of a sustainable lifestyle at 8,000 kg per annum for one person [17]. To stay within planetary boundaries, the material footprint of an average European would be required to drop from 50,000 kg per year (approximate current average lifestyle footprint per person) to 8,000 kg per year. The material footprint of 8,000 kg per annum consists of household goods, food and beverages, everyday mobility and tourism, electricity, heating and housing. The composition of an 8,000 kg footprint lifestyle is not similar for everyone. The share of consumption in a material footprint of 8,000 kg per annum can differ based on the values, needs and aspirations of each person’s unique lifestyle. An example of an 8,000 kg sustainable lifestyle might include (but is not exclusive to): the use of public transportation as primary source of mobility; a predominantly vegetarian diet (less meat, not no meat); a zero net-energy home; energy sourced from renewables; fewer and more efficient household appliances; and staycations as an alternative to long-haul leisure travel. The SPREAD Sustainable Lifestyles 2050 scenarios aim to help us to visualise what 8,000 kg living can look like in four diverse future societies to inspire options for change [18].

♦ *Challenge 5: recognising citizen movements*, *social innovation and promising practice.* We have found a diversity of promising sustainable living examples that can be categorised by three dominant trend areas: efficient lifestyles, different lifestyles and sufficient lifestyles. These trend areas have also been identified in previous work on sustainable consumption [19-22]. Efficient lifestyles refer to current household behaviour trends towards wasting less, including the more efficient choice, use and disposal of products and services. Different lifestyles refer to a shift in preferences from quantity and ownership to access and quality, the latter so-called Collaborative Consumption [23] – a shift in how we live, move and consume. Different living offers the potential to decouple material consumption from resource use. Sufficient lifestyles refer to the focus on improving quality of life and conscious efforts to consume less – as our lives become more complex, a growing number of people prefer to shape their lives in a simpler way to reduce the pressure created by an overabundance of stuff or to reduce the adverse impacts of overconsumption.

Policy, industry and civil society can play important roles in fostering and accelerating the pathways to scale sustainable lifestyles to mass-market adoption. Resilient change requires enabling environments and infrastructures to support long-lasting behaviour change [13]. Policy incentives and investments in the mass-market adoption of current promising sustainable living innovations – products, services and social innovation – can be important drivers of change. We seek to express optimism for the prospects of a future of more sustainable living for all. Our challenge lies in seizing the opportunities fast enough to bring about resilient change.

## Session 2: theory into practice in behaviour change

Changing consumer behaviour requires a portfolio of scientific techniques and methodologies. The second session demonstrated how multidisciplinary science can be deployed in designing, implementing and measuring theory-based behavioural change interventions. The session opened with Professor C Fergus Lowe describing the science behind behaviour change and practical solutions. Dr Robert J Crawford then described some examples of applied behaviour change, created through environmental modifications. The session concluded with BV Pradeep describing Unilever’s new applied model for behaviour change: Five Levers for Change.

### The science of behaviour change *Professor* C *Fergus Lowe*

Behaviour change appears to be an idea whose time has come. This has come about because modern market economies have given rise to a range of health and social problems that have proved impervious to traditional approaches of legislation, education and exhortation. Numbered among these problems are education systems that are failing; parents who lack the basic skills to bring up children; high rates of antisocial behaviour; obesity; alcoholism; drug abuse; the ongoing degradation of the planet; and, of course, the chaos that is the international banking and finance system. Most of these problems are, however, preventable and, because they are fundamentally behavioural problems, the solutions to them lie in behaviour change.

The interest in the behavioural change approach now being shown by governments and other agencies across the globe has been sparked by a number of recent books, such as Thaler and Sunstein’s *Nudge *[24] and Cialdini’s *Influence *[25], which have brought together a range of findings from experimental psychology and behavioural economics showing how behaviour can be nudged by environmental factors in ways that promote good health and well-being. For example, we are influenced by social norms to behave as we think most other people do, so that if we are told that lots of people do *x*, then we tend to do likewise; we are more influenced by people we like or who have authority; we have a tendency to be consistent with what we say we are going to do; and our behaviour is influenced by environmental prompts.

While the evidence for the influence of such factors is undeniable, there remains the question of whether the nudge approach is sufficient to deal with the complex and deep-rooted health and social issues that are of most concern to us. Solving these issues may require more than mere nudges. This presentation proposes that, if we are to achieve really sustainable behaviour change, we need to have powerful interventions that draw upon a systematic behavioural analysis of particular problems, and we need to develop integrated programmes that incorporate the many behavioural principles and processes we know will contribute to these programmes’ effectiveness.

Take, for example, the biggest public health problem of our time – obesity. Most often, obesity is viewed from medical and nutritional perspectives. Obesity is, however, fundamentally a behavioural problem. To combat obesity, people need to change their eating habits and their levels of physical activity. To prevent obesity occurring at all, habits should be altered early in childhood, which is when the disorder is set in motion [26,27].

Such was the approach we adopted in the development of a systematic behaviour change healthy-eating programme known as the Food Dudes [28,29]. Designed for children aged 2 to 11 years old, the programme brings together a range of behavioural influences.

In school, the children watch DVD films of the exploits of the four heroic Food Dudes – older children who love eating fruit and vegetables and who are in battle with General Junk and his Junk Punks. The films incorporate a range of social-norming and role-modelling principles [30,31].

In addition, when the children begin to taste the fruit and vegetables they think they do not like, they receive small Food Dudes prizes. The power of incentives has often been overlooked in this domain, but research shows that tangible rewards are crucial in bringing about large and sustainable behaviour change [32-34].

The design of the programme is such as to also ensure that the children have to repeatedly taste the same fruits and vegetables, because research shows that repeated tasting of particular foods leads to increased liking of them [35-37]. The programme also has many other features – in all, more than 50 behavioural principles and processes – that work in combination over time to bring about sustained behaviour change.

Evidence from several studies shows that the Food Dudes programme brings about large and long-lasting changes in children’s consumption of fruit and vegetables, often increasing it by more than 100%; that these effects are greatest in children who initially eat least of these foods; and that the effects generalise from school to home environments with not just the children but their parents also eating more fruit and vegetables [31,38-40].

The programme is designed to be scalable and to be effective for children in any country. Food Dudes has been introduced nationally by the Irish Government, so far, to more than 300,000 children in Irish primary schools. Rollout in the UK has begun with schools in the Midlands and other regions, involving 100,000 children. Successful pilot projects have also been conducted in the USA and Italy [34,41]. The significance of this work has also received recognition in the form of awards from prestigious bodies, which include, for example, an award from the World Health Organization and the UK’s Chief Medical Officer Gold Medal.

Whether or not this particular programme turns out to be the most effective intervention in the arena of obesity, what this presentation proposes is that this kind of systematic behaviour change intervention is not a mere option, but is essential to enable us to deal with serious and deep-seated behavioural issues of this nature. The science of behaviour change will provide the foundation for this endeavour.

### Domestic infrastructure: catalyst for behaviour change *Dr Robert J Crawford*

The physical living circumstances of people drive their daily habits and determine the limitations of behaviour, including the consumption of household and personal care products, which can have a positive influence on health and hygiene. As such, consumer companies like Unilever can usefully work with different public and private sectors to create change in living conditions to produce positive behaviour change and eventual better health. Unilever is currently pursuing three projects of relevance to this area: solar heated showers in South Africa, improved cookstoves in Kenya, and a sanitation service business in Ghana. In each case a significant improvement is made to the physical conditions of the house, and as a consequence positive behaviour change is created. These projects will help to understand commercially scalable models that create sustained positive behaviour change. Some important considerations common to all three projects are presented that underpin the approach.

Firstly, the recognition that Unilever’s core capability – what we can bring to the party – is branding and marketing. Consumer goods brands, including those of Unilever, are highly visible in developing markets. The assumption underlying this approach is that Unilever branding has a value in adjacent sectors, and that useful business models are possible based on the licensing of Unilever brands to other sectors (cooking stoves, solar showers, and sanitation in the examples presented). Unilever will expect a commercial return on such a deal – in part a royalty, in part an increase in sales of Unilever products.

Secondly, the use of Unilever branding and marketing in adjacent sectors requires that a link is made in the consumer’s mind between the consumption of Unilever products and the products and services of the other sectors (for example, stoves, warm water and showers, sanitation). This link provides the essential commercial logic to drive the collaboration across sectors. This link can take the form of promotional offers – for example, rewarding people’s loyalty to Unilever brands, or trial of new products, with access to improved facilities courtesy of other sectors (for example, stoves) – Sustainable Living Points, perhaps. For the other sectors, the association with Unilever brands provides much greater awareness and sales than they can achieve for themselves.

Thirdly, it is critical to understand what type of collaboration is necessary to develop and test such business models, and then to commercialise them. Multiple parties are involved, depending on the nature of the project. Each has their own objectives, so it can sometimes take some time and experimentation to find the best mutually beneficial models. This is the essence of the work – creating new relationships for mutual advantage and positive social and environmental impact. For example, Unilever South Africa has partnered to install over 6,000 solar water heaters in low-income houses, incorporating a carbon-credit scheme. These have an immediate impact on behaviour (people take more baths!). We then took 50 of these houses with solar water heaters and installed low-cost showers. First results again show an immediate impact on behaviour, as people love to take showers, but also a decrease in water consumption – to be confirmed over a longer period, but perhaps simply a result of the shift from buckets/basins/tin baths to low-flow showers. Current work is to understand the commercial basis – the brand licence and promotional campaign – that takes the model to scale. This basis might, for example, take the form of people collecting Sustainable Living Points from Unilever products in order to qualify for the solar shower scheme.

In Kenya, Unilever is partnering with Shell Foundation and their indoor air-pollution campaign to see how their local food brand can help bring an improved cookstove to every house. The benefits in terms of health through reduced indoor air pollution are very significant, together with reduced charcoal consumption (deforestation) and greater convenience. Again the task is to figure out the promotional link and commercial basis for licensing deals.

In Ghana, Unilever is investigating the power of branding to create a high-quality sanitation service business that also includes a direct sales element for consumable products. The objective is to create a profitable sanitation service that therefore has the potential to achieve scale – something that has hitherto proved very difficult to achieve in the sanitation sector. The profitability comes from people’s willingness to pay – which in turn comes from the introduction of aspiration (Unilever’s expertise) into an otherwise rather functional sector.

In each case it is important to learn and to experiment, and to do so in the field – getting direct feedback from consumers and partners along the way. The projects described are supported by Unilever Ghana, Unilever South Africa and Unilever Kenya – their support is fundamentally important to making progress and building new insights and models. Also critical is the support of Unilever’s many civil, commercial, municipal and academic partners, to whom great thanks are due.

### Applied behaviour change model: Five Levers for Change *BV Pradeep*

Unilever has a long history in improving health and the use of marketing and market research to promote behaviour change. In November 2011, for the first time, we published our own model of effective behaviour change: Unilever’s Five Levers for Change. This is a practical tool with a coherent set of principles, which, if applied consistently to behaviour change interventions, will increase the likelihood of having a lasting impact.

#### Overview of the five levers

The first step of the five levers model is to systematically mine consumer understanding to identify the key barriers (what are the things that stop people from adopting a new behaviour?), triggers (how could we get people to start a new behaviour?) and motivators (what are the ways to help them stick with the new behaviour?).

Next, we take all those insights and consider how to inspire change, using each of the Five Levers for Change:

♦ *Make it understood.* Do people know about the behaviour? Do they believe its relevance? This lever raises awareness and encourages acceptance. A good example of how this lever has been applied is the Unilever salt calculator. This calculator is a simple online diagnostic tool to make people aware of the amount of salt in their diet and products that may be significantly contributing to it. Likewise, Lifebuoy’s innovative Glo-Germ demo is a good diagnostic tool that overcomes the key mindset barrier of ‘I wash my hands with water and it looks clean. So, why use soap?’, by showing how washing with water alone, is not good enough. The tool does this by making visible the glowing particles that will not go away with water alone, but need soap.

♦ *Make it easy.* Do people know what to do and do they feel confident doing it? This lever establishes convenience and confidence; for example, by designing appropriate packs that enable people to adopt new habits. The cap of Small & Mighty concentrated liquid was designed to prevent people from dosing the same amount of liquid as a conventional dilute detergent. The cap optimised dosing for great cleaning results and also enhanced value-for-money perceptions.

♦ *Make it desirable.* This lever is about self and society. We tend to emulate the lifestyles and habits of people we respect. We like to follow the norms in society. So will this behaviour fit with how people relate to others? Will it fit with their actual or desired self-image? In many water-scarce countries, rinsing lather during hand laundry leads to a lot of water wastage. Comfort One Rinse fabric conditioner requires only one bucket for rinsing rather than three. This new one-rinse habit can make consumers feel like they are cutting corners. However, role-models such as Indonesia’s First Lady rinsed with one bucket to show this was an important habit to adopt.

♦ *Make it rewarding.* This lever focuses on the need to provide proof that it works and demonstrates the payoff for them. Do people know when they are doing the behaviour right? Do they get some sort of reward for doing it? Unilever’s haircare brand Suave in North America partnered with Walmart to encourage people to ‘Turn off the Tap’ whilst lathering hair in the shower. Showing consumers that electricity savings can be up to $100 a year, was a compelling reward for behaviour change rather than just doing it for the environment.

♦ *Make it a habit.* Once people have made a change, making the habit stick is difficult. This lever is about reinforcing and reminding. Lifebuoy soap’s handwashing campaigns run over a minimum of 21 days to encourage repetition of behaviour in relevant settings, every day. During each day of the programme, comic books, posters, quizzes and songs are used in activities designed to deliver the handwashing message, repeatedly in an engaging and memorable way. Compliance is also tallied on a daily sticker chart, with the help of mum and teachers, to reinforce the behaviour. It is crucial that the triggers and reinforcing messages stay in place for an extended period, even after this 21-day period.

The Five Levers for Change offer a coherent approach to thinking about behaviour change and putting it into practice. The model is not intended as a step-by-step process – but what we have learnt is that the most effective programmes apply all of the levers in some way.

#### Five Levers for Change: the way forward

We have used the five levers model across a number of different categories, brands and behavioural challenges. The result is some key learnings that are worth considering. The first is that behaviour change should never be positioned as a compromise to current behaviour, because this will never work for a consumer. For example, telling someone to eat something that is not as tasty just because it may be good for them will not work. The situation has to be win–win. Secondly, behaviour change requires an upfront and long-term investment – it could take well over the 3 to 5 years expected in business models to pay back. The model involves making behaviour the norm, demonstrating the benefits, reinforcing and reminding people to keep going. Thirdly, innovation and technology need to support designs that encourage change behaviour. For example, designing the right-size bottle cap for the concentrated liquid detergent encourages the right dosage. Developing compelling messages and delivering through multiple touch points (digital, in-store, text, television, and so on) can help embed new behaviours.

## Session 3: role of collaborations in behaviour change

The first two sessions of the symposium set the scene for the behaviour change challenge and showed the science and practical applications that can be used to achieve behaviour change. However, it is also apparent that behaviour change cannot be achieved in isolation. Changing population behaviour in regards to diet and nutrition, lifestyle, hygiene and sustainability is difficult and complex. One of the most challenging parts of this process is reaching the entire breadth of the population. This step requires scaling up efforts and sharing expertise, which can better be achieved through private–public partnerships. As such, this session commenced with the presentation of two success stories in behaviour change for better health, which were both derived within a scientific framework. Professor Ibrahim Elmadfa and Dr Gerda Feunekes presented on a sodium reduction strategy, and Dr Val Curtis and Dr Myriam Sidibe presented on a global handwashing campaign. The session concluded with Dr Marti van Liere discussing the need for collaborations between industry, governments, the private sector and nongovernmental organisations in order to create effective behaviour change.

### New approaches to sodium reduction: International Union of Nutritional Sciences and Unilever collaboration *Professor Ibrahim Elmadfa and Dr Gerda Feunekes*

Unilever and the International Union of Nutritional Sciences (IUNS) participate in a formal partnership aimed at reducing NCD, with a current focus on increasing demand for sodium-reduced foods, and generally lowering sodium intake. Sodium intakes around the globe are on average two to three times higher than that recommended by the World Health Organization and other health authorities [42]. Overconsumption of sodium is a major contributor to cardiovascular disease as it progressively raises blood pressure levels with age. Globally, an estimated 49% of coronary heart disease events and 62% of strokes can be attributed to elevated blood pressure [42]. In countries where effective sodium-reduction programmes were implemented, the prevalence of blood pressure and cardiovascular diseases decreased in parallel [43].

In industrialised countries, most of the sodium intake is derived from processed foods and restaurant foods. In developing countries, sodium is mainly added during food preservation and preparation, either as salt, seasonings or sauces. As taste preferences for salty foods are often linked to traditional food preservation and preparation, there is an opportunity for food industries to apply modern technology to help people reduce their sodium intake. However, general awareness on the need to reduce sodium intake is low and, furthermore, even if people are interested in reducing their sodium intake they need to be motivated to gradually adjust their salt preference by actively choosing sodium-reduced foods.

In light of this, in 2012 Unilever and the IUNS organised a series of six sodium-reduction behaviour change workshops with local public health experts and other relevant stakeholders, to generate ideas and actionable strategies to support behaviour change initiatives to reduce sodium intake amongst the general population. These workshops were conducted in Germany/ Austria, Hungary, South Africa, China, India and Brazil. Given the limited information available on this topic, a series of studies were performed to generate quantitative information on barriers and triggers for sodium reduction, as well as preferred sources and channels for promoting sodium reduction, as input for the workshops.

Based on two pilot workshops in the UK and the Netherlands, Unilever developed a format for the behaviour change workshops. The setup of the single-day workshops includes a ‘step in the shoes of consumers’ role-play exercise, where workshop participants first role-play a specific consumer in a focus group to work through a series of questions on salt by guessing how consumers would answer and experience such questions. Workshop participants then watch an actual focus group discussion of general consumers to see what the consumer reactions and experiences really were. Through engaging in such an exercise, participants generally grow to accept that education alone is not enough to drive consumer choice. This activity was then followed by a split-group structured brainstorm exercise, using Unilever’s Five Levers for Change (explained in the previous section), aimed at developing ideas for new sodium-reduction approaches. A cartoonist is present to help capture ideas and campaigns. The workshops are then concluded with a mediated session where the brainstorm ideas are shared, and concrete actions are captured.

The research studies consisted of consumer cohort studies in each of the aforementioned countries. Within each country a population representative sample of 1,000 adults (aged 18 to 65) filled out an online questionnaire, which was adapted to local language, foods and habits. The overall results revealed that, although salt reduction was an important topic and relevant to health, the majority of the respondents believed their sodium intake was satisfactory and they were not thinking of, or planning to, reduce their sodium intake. Interestingly, South Africa and China were relatively advanced in terms of awareness of recommendations and intentions to reduce sodium intake, probably linked to recent local communication on the need to reduce sodium intake. Other key insights from the survey were that the perceived importance of low-salt food choices grew with an advanced stage of behaviour change awareness, and that people felt that they themselves were responsible for their salt intake, independent of the stage of change. Full results on each of the local surveys were provided as input to the local IUNS behaviour change workshops.

The completed workshops were reported as successful by the IUNS and Unilever representatives. These workshops, lead by IUNS, helped to engage public health stakeholders, ranging from nutrition experts to consumer organisations and food industry representatives, in jointly developing exciting behaviour change approaches in the area of salt reduction. They were only a first step of a local sodium-reduction journey, which will require follow-up meetings for implementing and upscaling of the agreed consumer-focused salt-reduction approaches. At a global level, output from the surveys and workshops will be used to identify common themes around the world, and cluster countries where similar approaches could work. Combining an increased availability of sodium-reduced foods with generic, public health-type campaigns and motivational, branded campaigns will increase consumer awareness on the need to reduce sodium intakes and will motivate consumers to try more sodium-reduced foods, while gradually adjusting their taste preference.

### Driving handwashing habit change at scale: one billion by 2015 *Or Valerie Curtis and Dr Myriam Sidibe*

Whilst many people in the developed world worry about overconsumption, at the same time there remains a silent emergency of underconsumption in many developing countries. Two out of five people on the planet still have no toilet, and some four out of five people still do not wash their hands with soap at key moments [44]. As a result, three-quarters of a million children lose their lives to diarrhoeal disease before they reach the age of 5 [45]. Soap is an affordable and readily available technology that could cut the diarrhoea risk almost by one-half [46,47] and could also prevent respiratory infections [48]. Although present in almost every home in developing countries [49], soap is still not used for handwashing at key times, especially after contact with faecal material and before feeding children. In this presentation we describe a body of work that combines new approaches to behaviour change with the market power and reach of Lifebuoy, a major soap brand, to bring handwashing with soap to the billions that need it most.

#### The importance of motives

In the field of health promotion, theories of behaviour abound. Health psychologists have most often used the Theory of Planned Behaviour [50], the Health Belief Model [51] or the Health Action Process Approach [52]. These theories are based on the assumption that behaviour is cognitive, conscious and calculated to produce rational outcomes. However, most behaviour is not under rational control but is driven by motives that cause people to seek out and secure the things they need to survive and reproduce effectively. These needs include food, mates, social bonds and social justice (Aunger R, Curtis V, manuscript submitted), and do not include health.

If rational appeals to behave in healthier ways are of limited use and there is no health motive, then how should we design handwashing campaigns? First, we must better understand the drivers of handwashing behaviour; and second, we need to translate our understanding into effective large-scale programmes.

#### Motivations behind handwashing

Studies of handwashing were carried out in over 12 mainly developing countries using the lens of the Evo–Eco model [49]. We used methods such as structured observation, behaviour trials, the elicitation of daily routines, video ethnography, projective techniques using stories and pictures and Internet-based questionnaires. One important finding was that, although there is some variation from country to country and from setting to setting, many of the basic drivers of handwashing behaviour were the same.

Habit was the most important psychological determinant of handwashing behaviour [53]. Behaviour trials showed that handwashing would only happen habitually if it was incorporated into the daily routine. This required preparing facilities and using place and previous activity to cue the behaviour. Many different motives for handwashing were explored, including status (high-class people/celebrities wash their hands) and attraction (your husband loves clean hands). However, the most effective were disgust (invisible contamination on your hands can only be removed with soap), nurture (your child will thank you/love you for the care you took to keep his/her hands clean) and affiliation (doing what everyone else is doing). Disgust has been shown to be effective in encouraging handwashing in a number of studies [54,55]. Also well established is the fact that affiliation is a key motive: changing local norms can do much to change behaviour [56]. The formative research brought to light the importance that mothers and schools attached to teaching children good manners, and that this might be employed in changing societal norms around handwashing.

#### Lifebuoy non-negotiables

Unilever’s Lifebuoy brand has committed to getting a billion people washing hands with soap by the year 2015. The results of the global formative research, along with local research and the Unilever Five Levers for Change, helped to provide the components of the current campaigns, which have already reached 48 million people in 2011 in countries including Indonesia, India, Vietnam, Bangladesh, Pakistan, Malaysia, Kenya and Ghana.

To ensure uniform delivery of the programme across so many countries, Lifebuoy developed five non-negotiables – core principles of the campaigns that came from theory and evidence and were to be adapted to local conditions. These non-negotiables were as follows:

♦ Disgust: in the interaction with the school children, in order to show that the use of soap is important, the Glo-Germ demonstration shows children that there is invisible contamination on their hands – this provides a visceral emotional experience that is more powerful than a lecture about germs.

♦ Nurture (mother and child interaction): mothers are encouraged to enforce the handwashing habit at home contributing to their perception of being a good mum – for new mums, the creative work is based on the idea that ‘Your child trusts your hands the most’,

♦ Affiliation (positive reinforcement): Lifebuoy created ‘the school of five’ where kids want to join the group, aided by a well-respected and loved celebrity.

♦ Habit (21-day practice): making soap use in the five occasions into a new habit means encouraging mothers and children to do the same – to repeat behaviours again and again in the same settings until they stick.

♦ Pledging and creating new norms: mothers and children take pledges in front of others whose opinions matter – making handwashing good manners and therefore socially desirable.

#### Conclusions

Whilst we increasingly understand the drivers of healthy behaviour, such as handwashing, huge challenges still remain in implementation. We know that approaches such as those described above can be effective at the small and medium scale [57,58]. However, with multiple millions of people still to reach, the priority now has to be finding ways of making such interventions as cheap as possible, and to anchor them into the education and health systems of every country. Every mother and every teacher should be entrenching the habit of handwashing with soap in their charges. Only then will handwashing become a norm for the majority of the population, and children in every country in all walks of life will be protected from fatal infectious illness.

### Importance of collaborations in changing behaviour *Dr Marti van Liere*

The Global Alliance for Improved Nutrition works with both public and private-sector partners to improve the accessibility and affordability of appropriately fortified foods in developing countries. The Global Alliance for Improved Nutrition has just celebrated its 10-year anniversary in mid-2012 and is now reaching over 660 million people with nutritious, fortified food products – such as iodised salt, vegetable oil enriched with vitamin A, or fortified complementary foods for babies 6 to 24 months old. As the Global Alliance for Improved Nutrition brought these high-quality fortified products to the market, they realised that consumer behaviour needed to be influenced so that they start choosing and using fortified food products.

Traditionally, infant feeding behaviour change campaigns, inspired by the World Health Organization infant feeding guidelines [59,60], have been executed by the health sector. Although projects have demonstrated an impact of interventions on behaviour and even nutritional status, there are not enough examples of well-documented, large-scale programmes that have successfully improved feeding practices in children 6 to 23 months old and have resulted in improved health outcomes at national level. Campaigns often transmit multiple, complex messages to illiterate mothers – leaving it to her to figure out how to find the time to breastfeed or the financial means to prepare a diverse diet especially for the baby. These messages ignore the reality of globalisation, urbanisation and modernisation: many of these mothers, although poor, are having long working days in rural and urban settings; they may have a cell phone but do not grow fresh produce in their own home garden or may not be able to access it at an affordable price at the wet market or supermarket. Mothers and caregivers not only wish for their children to be healthy and grow up well, they are also consumers, looking for convenience, making their choices based on the available products and information.

The public health sector can learn from commercial marketing to become more effective in driving the desired behaviour change. Where public health focuses on the needs of people for better health, hygiene and nutrition, translating this into well-intended knowledge messages, marketers understand that human behaviour is also driven by the wants of consumers, the more immediate benefits one can get out of life. Advertisement is based on behavioural psychology insights that trigger a consumer to purchase a product not because she/he needs it for a rational reason, but because she/he wants it for an emotional reason. Often this approach is more impactful and effective than a knowledge-based awareness-raising campaign.

Technological solutions to improve health, such as water purifiers, medication or fortified foods, will not have the desired health impact if there is no or limited uptake by the targeted users. Alliances such as Water and Sanitation for the Urban Poor, Scaling Up Nutrition and the CEO Water Mandate have recognised the value of multi-stakeholder collaboration, including the private sector, to scale-up impact in health, nutrition or environment at a country level.

Public health and nutrition experts should tap into the strengths of the commercial private sector, to achieve sustainable impact on a large scale in improving infant feeding behaviour and nutritional outcomes.

In multi-stakeholder collaborations, each stakeholder brings its own strengths to the table: academics lend credibility to the messages and provide the scientific evidence base; industry brings expertise in innovation, production, distribution and marketing; and public health services and nongovernmental organisations understand the needs of the poorest and have the capacity to reach the most vulnerable target groups. Policy-makers and regulatory authorities are also important players, establishing regulatory standards and marketing guidelines that allow or inhibit claims that are presumed to influence purchasing behaviour. The user or consumer should be central, as she/he is the person that will take an informed decision to make use or not of an innovation. Consumers are not just passive receivers but are key drivers of change, which is why not only their needs but, foremost, their wants should be central to all stakeholders.

Partnerships between the public and private sectors are not without challenges. One of the main hurdles is the negative perception of the business benefit by those working in the public sector: why should nongovernmental organisations or governments support companies to market their products and help them make profit? There is a thin line between demand creation for a product category or a generic habit (promotion of consumption of iodised salt) and the advertisement and promotion of a branded commercial product (use of a specific salt brand). From a business perspective, however, it makes sense that investments in a partnership are expected to bring a benefit and contribute to the business key performance indicators, such as brand equity, supply-chain efficiencies or product penetration or sales. Collaborations or partnerships are hard work and require adequate time and energy investment.

They can only work if the main principles are trust, transparency and equity. Success factors include the definition of a clear roadmap with shared goals and transparency about the individual goals (including business objectives), and measurable key performance indicators for each partner. Roles and responsibilities of each partner should be spelled out at the start. Partners must discuss a common communication framework including, if appropriate, guidelines for use of logo and branding. Many public-sector organisations have strict rules regarding non-endorsement of products or brands. Appointing a designated focal point is important, as is frequent and open communication, but senior leadership support is even more crucial. Only then will the partnership be allowed sufficient time and resources to develop and deliver.

Changing behaviour for better health requires passionate and creative marketers applying their skills and competencies to solve complex changes in behaviour to improve health and nutrition of underprivileged target groups. This change of behaviour also requires passionate and committed public health experts to create access to appropriate solutions and choices through multiple channels, and to empower vulnerable target groups to make informed decisions based on their own needs and wants.

There is no easy or quick fix to changing behaviour for better health or better nutrition, but the potential of achieving impact at scale through public–private sector collaborations makes the investment worthwhile.

## Abbreviations

NCD: noncommunicable disease; IUNS: International Union of Nutritional Sciences.

## Competing interests

VC, IE, CH, CFL, ML, KSR and RU received an honorarium for their contributions to the symposium and the current paper from Unilever Netherlands. RSN, RL, RJC, GIJF, GWM, BVP and MS are employees of Unilever. CFL is CEO of Food Dudes Health.

## Declarations

This article has been published as part of *BMC Public Health* Volume 13 Supplement 1, 2013: Behaviour change for better health: nutrition, hygiene and sustainability. The full contents of the supplement are available online at http://www.biomedcentral.com/bmcpublichealth/supplements/13/S1. Publication of this supplement was supported by Unilever R&D Vlaardingen. The meeting report is based on proceedings of the conference, was submitted by the sponsor and was fully peer reviewed by the journal. The Supplement Editor declares that they have no competing interests.
